# Tumor endothelium-derived PODXL correlates with immunosuppressive microenvironment and poor prognosis in cervical cancer patients receiving radiotherapy or chemoradiotherapy

**DOI:** 10.1186/s40364-024-00655-0

**Published:** 2024-09-18

**Authors:** Rui Huang, Fuhao Wang, Wenxue Zou, Xiaohui Li, Tianyu Lei, Peihang Li, Yajun Song, Chao Liu, Jinbo Yue

**Affiliations:** 1 Department of Oncology, Chongqing University Fuling Hospital, Chongqing, China; 2 Department of Radiation Oncology, Shandong Cancer Hospital and Institute, Shandong First Medical Universityand, Shandong Academy of Medical Sciences , Jinan, China; 3 Department of Oncology, Linyi Center Hospital, Linyi, Shandong China; 4 Department of Medical Oncology, Peking University First Hospital, Beijing, China; 5 Department of Oncology, Renmin Hospital of Wuhan University, Wuhan, China; 6 Department of Radiation Oncology, Tianjin Clinical Research Center for Cancer, Key Laboratory of Cancer Prevention and Therapy, National Clinical Research Center for Cancer, Tianjin Medical University Cancer Institute and Hospital, Tianjin, China

**Keywords:** Cervical cancer, Chemoradiotherapy, Immune response, Prognosis

## Abstract

Podocalyxin-like protein (PODXL) is known to originate from tumor cells in several cancers; however, which cell type it is expressed in, whether and how it may contribute to tumor progression after radiotherapy or chemoradiotherapy in cervical cancer (CC) remain unknown. In this study, we investigated these issues using a cohort of 180 immune stain data, single-cell RNA sequencing (scRNA-seq) data of 29,453 cells, and bulk RNA sequencing data from 187 cervical cancer samples treated with radiotherapy or chemoradiotherapy. ScRNA-seq analysis revealed that *PODXL* was predominantly expressed in tumor endothelial cells (TECs) of CC, which was corroborated by tumor section staining. Moreover, the PODXL expression level was negatively associated with progression-free survival and overall survival of 180 CC patients receiving radiotherapy or chemoradiotherapy (both *p* < 0.001). Furthermore, compared with *PODXL*^low^ TECs, *PODXL*^high^ TECs exhibited a diminished anti-tumor immune response and enhanced tumor-promoting features characteristics. In addition, *PODXL* over-expression was also found to be negatively associated with immune response and indicated poor survival in bulk RNA sequencing data of CC treated with radiotherapy or chemoradiotherapy. These results underscore the role of PODXL in CC, suggesting it as a promising target and prognostic marker for patients treated with radiotherapy or chemoradiotherapy.

**Supplementary Information:**

The online version contains supplementary material available at 10.1186/s40364-024-00655-0.

To the Editor,

Cervical cancer (CC) is one of the most common malignancies of the female reproductive system [1, 2]. For locally advanced stages of CC, chemoradiotherapy represents the standard therapeutic approach [3, 4]. However, approximately 23% of patients experience local or metastatic relapses following chemoradiotherapy [5], and the overall prognosis of which remains poor [6]. Therefore, it is crucial to identify novel biomarkers that can provide prognostic indicators for CC patients undergoing radiotherapy or chemoradiotherapy, potentially serving as targets for optimized combination therapies. The podocalyxin-like (PODXL) protein is reported to be overexpressed in tumor cells and plays an essential role in the tumor progression and metastasis in several cancers [7–11]. However, the role of PODXL in CC remains largely unknown, including the specific cell type in which it is expressed and whether it is associated with prognosis following radiotherapy or chemoradiotherapy, and how it may contribute to the progression of CC. In this study, we uncovered the distinct role of PODXL predominantly expressed in tumor endothelial cells (TECs) in CC, which differs from its role in other cancers and suggests that it could serve as a valuable therapeutic target and biomarker for CC patients receiving radiotherapy or chemoradiotherapy.

Our previous study performed single-cell RNA sequencing (scRNA-seq) on tissues spanned from normal cervix to advanced cervical squamous cell carcinoma, revealing a subset of endothelial cells with elevated *PODXL* expression, which displayed proliferative traits and reduced survival [12]. However, it remains unclear whether it is specifically expressed in TECs of CC receiving chemoradiotherapy rather than on cancer cells, as observed in other tumor types, and how it contributes to tumor progression. Thus, we analyzed the scRNA-seq data of 29,453 cells from 5 treatment-naive CC patients (Fig. 1A). Ten major cell populations were identified by known lineage markers with NK cells (*KLRB1*), T cells (*PTPRC, CD3E*), B cells (*MS4A1*), myeloid cells (*CD68*), plasma cells (*MZB1*), pDC (*IRF7*), CAF(*PDGFRB*), FAP^+^CAF (*FAP*), epithelial cells (*KRT19*), and TECs (*VWF*) (Fig. 1A and Fig. S1A). These cell clusters also exhibited characteristic transcriptional profiles with differentially expressed genes (DEGs) (Fig. S1B). Notably, we found that *PODXL* was predominantly expressed in TECs (Fig. 1B). To further validate the scRNA-seq results, we conducted immunofluorescent staining of CC tissue sections, which indicated the predominant expression of PODXL in TECs (Fig. 1C). In conclusion, the above results indicated that *PODXL* serves as a specific marker for TECs in CC.

**Fig. 1:**
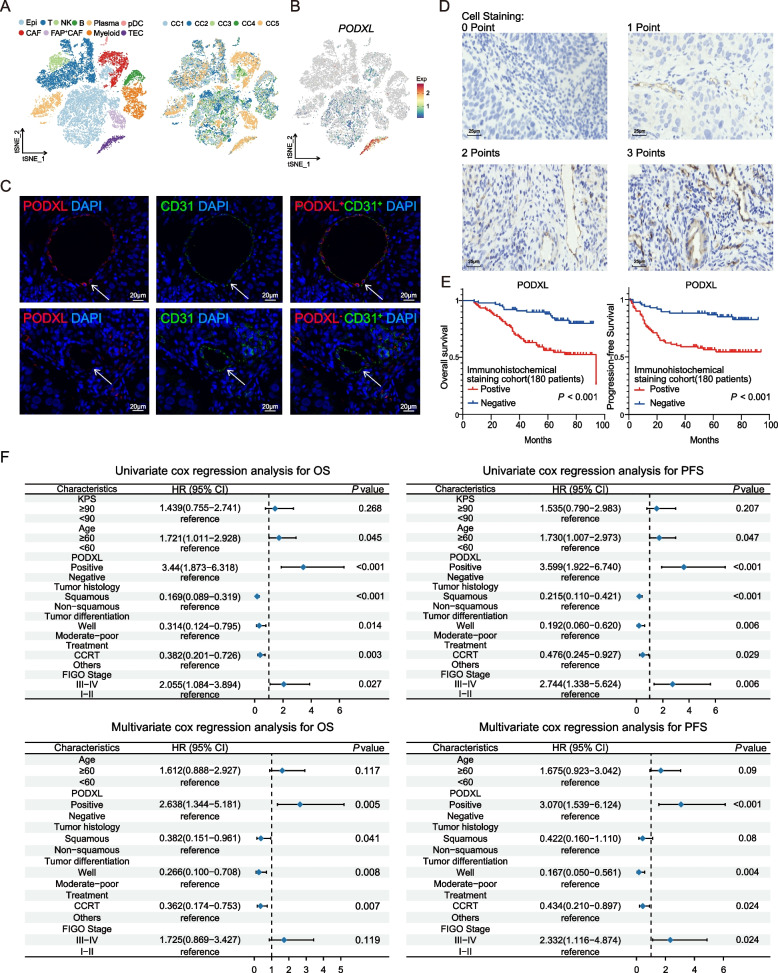
The predominant expression of PODXL in TECs and its prognostic value in CC patients treated with radiotherapy or chemoradiotherapy were revealed by our own immunostaining data from 180 CC patients and single-cell RNA-sequencing (scRNA-seq) data of 29,453 cells from 5 CC patients. **A** tSNE plots showing the whole 29,453 cells from scRNA-seq data, colored by cell type and samples origin. **B** tSNE plot illustrating the expression of *PODXL*. **C** Representative immunofluorescent labeling of PODXL (red) and CD31(green) for TECs in tumor sections from CESC samples (Scale bar, 20 μm). Top, positive PODXL expression in TECs; bottom, negative PODXL expression in TECs. **D** Representative immunohistochemical staining patterns of PODXL expression in TECs (Scale bar, 25 μm). Degree of cell staining: top left, no staining, 0 point; top right, yellow, 1 point; bottom left, brown, 2 points; bottom right, dark brown or black, 3 points. **E** Kaplan–Meier survival curves for OS (left) and PFS (right) in CC patients from our own cohort, stratified by positive and negative PODXL expression. The *p*-value of the two-sided log-rank test is shown. **F** The Forest plot showing the univariate analyses and multivariate analyses for OS (left) and PFS (right). CC: cervical cancer; scRNA-seq, single-cell RNA sequencing; tSNE: t-distributed stochastic neighbor embedding; TECs: tumor endothelial cells; PFS: progressive-free survival; OS: overall survival

Furthermore, we explored the relationship between PODXL expression levels and survival outcomes of CC patients treated with radiotherapy or chemoradiotherapy within our own cohort. A total of 180 CC patients who received these treatments were enrolled to form the immunohistochemical staining cohort (Fig. S2). A schematic representation of various expression levels of PODXL in CC patients was shown in Fig. 1D. Kaplan–Meier survival curves for this cohort revealed that positive PODXL expression was significantly associated with poor overall survival (OS) and progressive-free survival (PFS) in CC patients with radiotherapy or chemoradiotherapy (both *p* < 0.001; Fig. 1E). In this cohort, univariate Cox proportional-hazards model analysis showed that positive PODXL expression, age, tumor pathological type, tumor cell differentiation, tumor stage according to the 2018 FIGO staging system, and treatment regimen were significant predictors of OS and PFS in CC patients receiving radiotherapy or chemoradiotherapy (Fig. 1F). These statistically significant variables were subsequently included in the multivariate Cox proportional-hazards model analysis, which identified positive PODXL expression, degree of tumor cell differentiation and treatment strategy as significant predictors of PFS in CC patients who underwent radiotherapy or chemoradiotherapy (all *p* < 0.001, Fig. 1F).

To investigate how PODXL promote the progression of CC, we further analyzed these TECs in the scRNA-seq data and divided them into two groups (*PODXL*^high^ TECs and *PODXL*^low^ TECs group) based on the level of *PODXL* expression (Fig. 2A). The two groups of TECs exhibited distinct transcriptomic profiles (Fig. S3). For example, the *PODXL*^high^ *TEC*s group highly expressed *SLC9A3R2, FLT1* and *TIMP3* genes, while the genes upregulated in the *PODXL*^low^ TECs group included *ACKR1, MMRN1* and *SELP* (Fig. 2B). Notably, compared with *PODXL*^low^ TECs, the *PODXL*^high^ TECs exhibited higher tumor-promoting characteristics and poorer anti-tumor immune response, evidenced by the upregulation of angiogenesis, endothelial cell development and migration, and epithelial cell differentiation and migration pathways, and the downregulation of immune-related features including antigen presentation and processing, interferon production, and the T-cell activation and B-cell mediated immunity pathways (Fig. 2C-D and Fig. S4). Trajectory analysis of endothelial cell further showed the differentiation from *PODXL*^high^ TECs to *PODXL*^low^ TECs, accompanied by the downregulation of angiogenesis-related genes such as *FLT1*, *ESM1* and *KDR* (Fig. S5). In addition, the cell interaction analysis revealed that the epithelial cells exhibited more interactions with *PODXL*^high^ TECs than with *PODXL*^low^ TECs, particularly through the VEGF signaling pathway, promoting endothelial development and angiogenesis (Fig. S6).

**Fig. 2:**
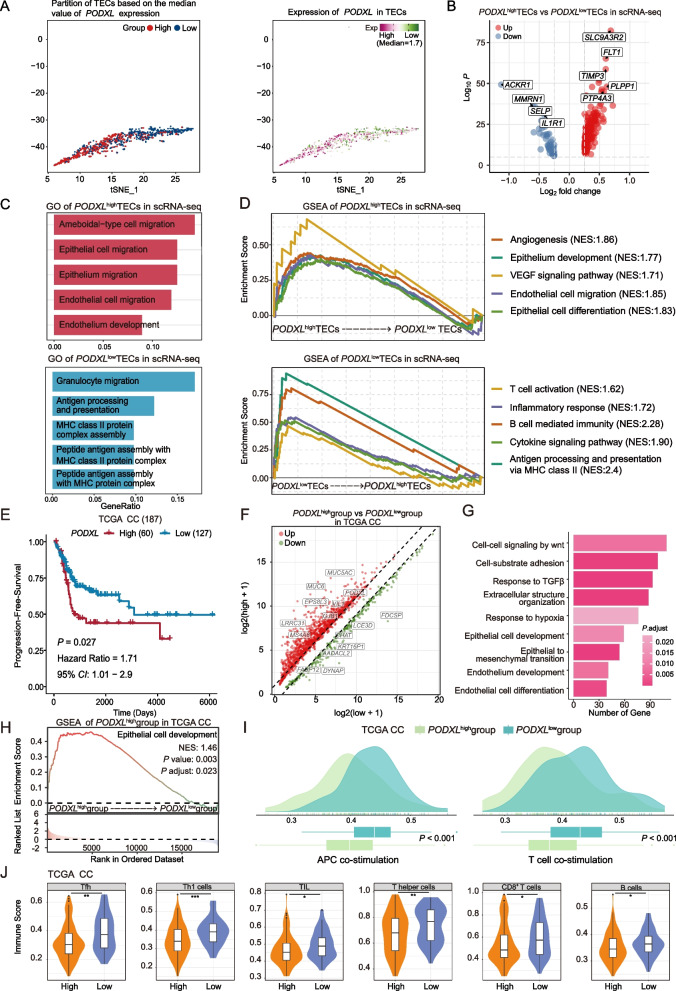
The characterization of high *PODXL* expression in CC patients treated with radiotherapy or chemoradiotherapy based on scRNA-seq and bulk RNA-seq data. **A** tSNE plots showing the 939 TECs, colored by *PODXL* expression and groups stratified by the *PODXL* expression (with the median cutoff of 1.7). **B** The volcano plot showing the DEGs between *PODXL*^high^ TECs and *PODXL*^low^ TECs in scRNA-seq data. **C** GO and **D** GSEA analysis of scRNA-seq data indicating the upregulated and downregulated biological processes and pathway activities in *PODXL*^high^ TECs, all of which exhibited statistically significant enrichment at *p.*adjust < 0.05. **E** Kaplan–Meier survival curve for progression-free survival of TCGA CC patients underwent radiotherapy or chemoradiotherapy, stratified by high and low *PODXL* expression (with the optimal cutoff value assigned). The *p*-value of the two-sided log-rank test is shown. **F** Volcano plot showing the differentially expressed genes between the *PODXL*^high^ and *PODXL*^low^ expression CC groups. The colored dots represent the top most variable genes. **G** GO analysis showing the enriched pathway in the *PODXL*^high^ CC group, *p.*adjust < 0.05. **H** GSEA analysis showing the enriched pathway of regulation of epithelium development pathway in the *PODXL*^high^ group, *p.*adjust = 0.023. **I** Density plots showing differences in APC co-stimulation score and T cell co-stimulation score between *PODXL*^low^ and *PODXL*^high^ CC groups (Wilcoxon test). **J** Violin plot showing the levels of Tfh, Th1, TIL, T helper, CD8^+^ T and B cells between the *PODXL*^low^ and *PODXL*^high^ CC groups. *, *p* < 0.05; **, *p* < 0.01; ***, *p* < 0.001 (Wilcoxon test). APC, antigen presenting cell; bulk RNA-seq, bulk RNA sequencing; CC: cervical cancer; DEGs, differentially expressed genes; GO: Gene Ontology; GSEA: Gene Set Enrichment Analysis; NES: normalized enrichment score; scRNA-seq, single-cell RNA sequencing; TCGA: The Cancer Genome Atlas; TECs, tumor endothelial cells

To further validate the role of PODXL in CC patients treated with radiotherapy or chemoradiotherapy, we employed bulk RNA-seq data from 187 CC patients who underwent radiotherapy or chemoradiotherapy, sourced from the TCGA database. Survival analysis showed that CC patients with high *PODXL* expression (*n* = 60) displayed a poorer prognosis following radiotherapy or chemoradiotherapy (HR = 1.71, 95%CI = 1.01–2.9, *p* = 0.027; Fig. 2E). Then, we performed DEGs analysis between *PODXL*^high^ and *PODXL*^low^ group, and found 1536 up-regulated and 338 down-regulated DEGs in *PODXL*^high^ group (Fig. 2F). The further gene ontology enrichment analysis of 1536 up-regulated DEGs revealed that the pathways of promoting epithelial cell proliferation were enriched in the *PODXL*^high^ group (Fig. 2G). Meanwhile, the gene set enrichment analysis validated that the *PODXL*^high^ group was significantly enriched with the regulation of epithelium development and other pathways promoting tumor progression (Fig. 2H and Fig. S7). In addition, genes associated with epithelial proliferation, migration, and invasion pathways were expressed at higher levels in the *PODXL*^high^ group than the *PODXL*^low^ group (Fig. S8A). This finding was further corroborated by our cohort of 50 CC patients, where we observed that the group with high *PODXL* expression exhibited lower degrees of pathological differentiation (Fig. S8B). Furthermore, Ki67 immunohistochemical staining revealed that the *PODXL*^high^ group had a significantly higher percentage of cells with Ki67 positive expression, further suggesting the role of PODXL in promoting tumor proliferation (Fig. S8C). It is also important to acknowledge the limitation that further functional experiments are needed to validate the tumor-promoting characteristics of PODXL^high^ TEC subsets in our future research.

Finally, we evaluated the immune infiltration between the two groups and the results indicated that the *PODXL*^low^ group exhibited higher antigen presentation cell and T cell co-stimulation density score (both *p* < 0.05; Fig. 2I). The scores of immune cells (Tfh, Th1, TIL, Th, CD8^+^T, and B cells) in the *PODXL*^high^ group were also lower in the *PODXL*^high^ group (all *p* < 0.05; Fig. 2J; Fig. S9). Altogether, our findings revealed that overexpression of PODXL was negatively associated with immune response and indicated poor survival in CC patients receiving radiotherapy or chemoradiotherapy.

In conclusion, the expression of PODXL in TECs plays a significant role in determining the prognosis of patients with CC treated with radiotherapy or chemoradiotherapy. We demonstrated that PODXL, associated with poor prognosis, was specifically expressed in TECs in CC and we also delved into its underlying features, offering new insights into its significance in cancer progression. Therefore, PODXL could emerge as a crucial prognostic marker and therapeutic target in CC patients undergoing radiotherapy or chemoradiotherapy.

## Supplementary Information

Supplementary Material 1:  Figure S1. The identification of cell clusters. (A) tSNE plots showing the marker genes expression for cell type identification. The legend shows a color gradient of normalized expression. (B) Heatmap showing the top five differentially expressed genes of each cell cluster. The intensity of the color indicates the average expression of the genes. tSNE: t-distributed stochastic neighbor embedding.

Supplementary Material 2: Figure S2. The baseline characteristics of the 180 patients comprised the immunohistochemical staining cohort.

Supplementary Material 3: Figure S3. Heatmap showing the differentially expressed genes between PODXL high TECs and PODXL low TECs in scRNA-seq data. TECs, tumor endothelial cells; scRNA-seq, single-cell RNA sequencing.

Supplementary Material 4: Figure S4: Gene set variation analysis revealed the comparation of tumor pathways between the PODXL low and PODXL high TECs in scRNA-seq data. ***, *p*  < 0.001 (Wilcoxon test).

Supplementary Material 5: Figure S5. Pseudotime analysis of PODXL low and PODXL high TECs in scRNA-seq data. (A) Three trajectory plots showing the predicted order of cell differentiation, pseudotime, and the expression levels of PODXL . (B) Heatmap showing the dynamic expression patterns of different genes along the pseudotime trajectory. Genes are categorized into four distinct expression patterns, marked by different colors.

Supplementary Material 6: Figure S6. Cell communication analysis of PODXL low and PODXL high TECs with epithelial cells. (A) Differential interaction network illustrating the number of interactions and interaction strength between PODXL low and PODXL high TECs and epithelial cells. The numbers indicate the count of differential interactions/strength. (B) Bar charts of interaction metrics. Left: Number of inferred interactions. Right: Interaction strength. (C) Heatmap showing the importance of different cell roles (Sender, Receiver, Mediator, Influencer) in the VEGF signaling pathway. Darker green indicates higher importance. (D) Heatmap showing the maximum communication probability for different VEGF ligand-receptor pairs. Columns represent the direction of communication. (E) Violin plots showing the expression levels of VEGF ligands and receptors. VEGF: Vascular endothelial growth factor.

Supplementary Material 7: Figure S7. The feature of PODXL high CC groups in TCGA database. Gene set enrichment analysis showing the enriched pathways in the PODXL high group. NES: normalized enrichment score.

Supplementary Material 8: Figure S8. Analysis of PODXL expression in relation to epithelial cell differentiation and migration. (A) Box plots displaying the expression levels of genes associated with epithelial proliferation, invasion and metastasis in PODXL high and PODXL low groups from the TCGA dataset. (B) Percentage bar chart showing the distribution of differentiation degrees (Low, Low-Middle, Middle, High) in our clinical cohort of 50 patients stratified by PODXL expression levels. (C) Immunohistochemistry analysis of Ki67 expression, comparing the percentage of Ki67-positive cells in PODXL high and PODXL low groups, with representative images and quantification. *, *p*  < 0.05; **, *p*  < 0.01; ***, *p*  < 0.001 (Wilcoxon test).

Supplementary Material 9: Figure S9. The difference of immune infiltration between the PODXL low and PODXL high CC groups in TCGA database. *, *p*  < 0.05; **, *p*  < 0.01; ***, *p*  < 0.001 (Wilcoxon test).

## Data Availability

The data described in this article can be freely and openly accessed at Genome Sequence Archive: 10.1126/sciadv.add8977. Additional resources used in this study can be requested from the corresponding authors upon reasonable request.

## Abbreviations

PODXL: Podocalyxin-like
CC: Cervical cancer
ScRNA-seq: Single-cell RNA sequencing
TECs: Tumor endothelial cells
OS: Overall survival
PFS: Progressive-free survival
DEGs: Differentially expressed genes
