# Supplementary figures and images for: Tumor endothelium-derived PODXL correlates with immunosuppressive microenvironment and poor prognosis in cervical cancer patients receiving radiotherapy or chemoradiotherapy
Source: Biomark Res. 2024 Sep 18;12:106. doi: 10.1186/s40364-024-00655-0 (PMC11409751; doi:10.1186/s40364-024-00655-0)

A

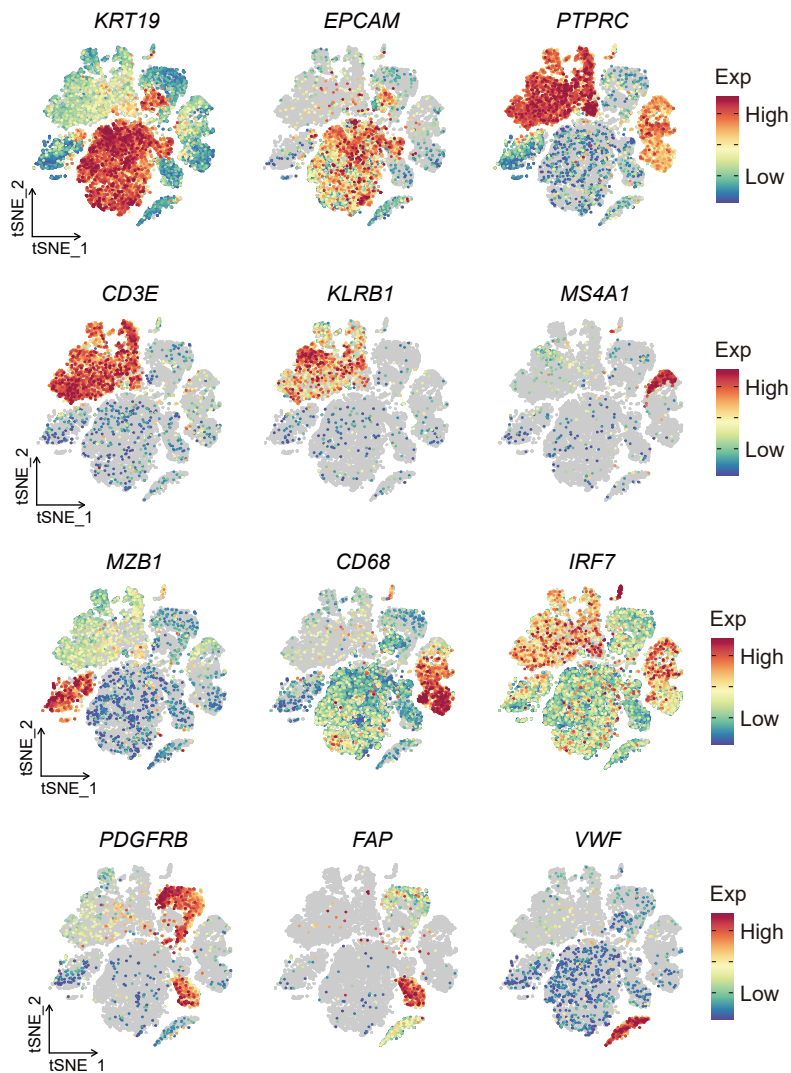

B

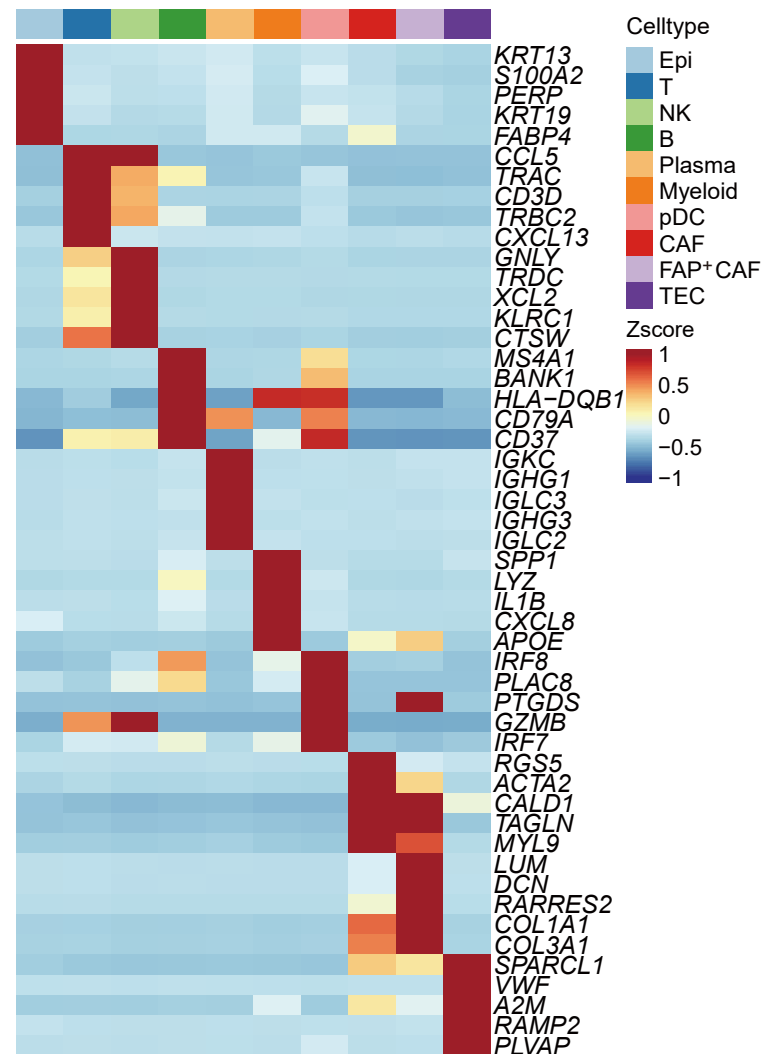

Supplement: Supplementary file 1 — Supplementary Material 1: Figure S1. The identification of cell clusters. (A) tSNE plots showing the marker genes expression for cell type identification. The legend shows a color gradient of normalized expression. (B) Heatmap showing the top five differentially expressed genes of each cell cluster. The intensity of the color indicates the average expression of the genes. tSNE: t-distributed stochastic neighbor embedding. [file 40364_2024_655_MOESM1_ESM.pdf]

PODXL

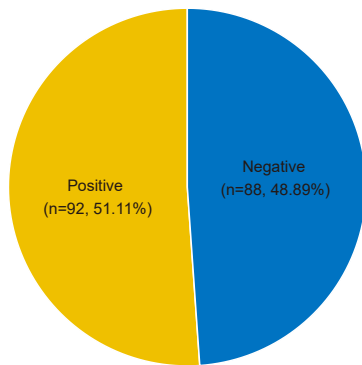

Age (years)

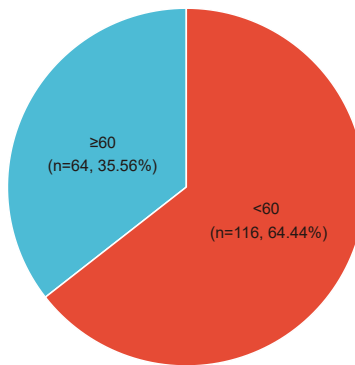

KPS

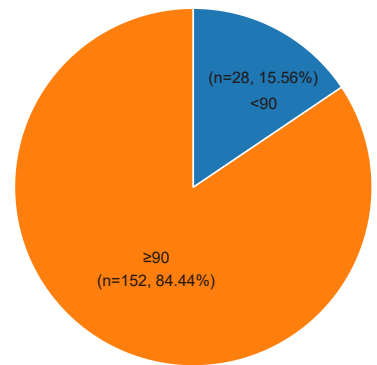

Treatment strategies

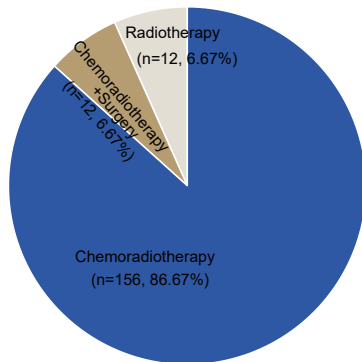

Tumor histology

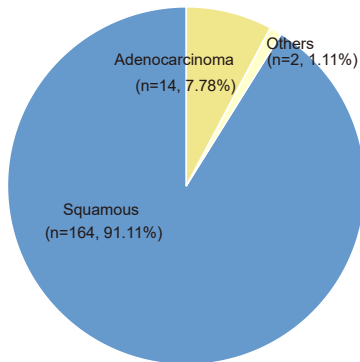

FIGO Stage

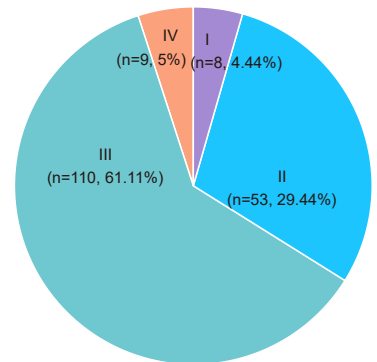

Tumor differentiation

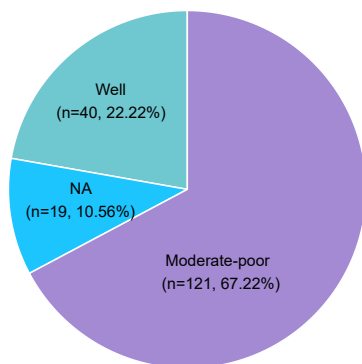

Supplement: Supplementary file 2 — Supplementary Material 2: Figure S2. The baseline characteristics of the 180 patients comprised the immunohistochemical staining cohort. [file 40364_2024_655_MOESM2_ESM.pdf]

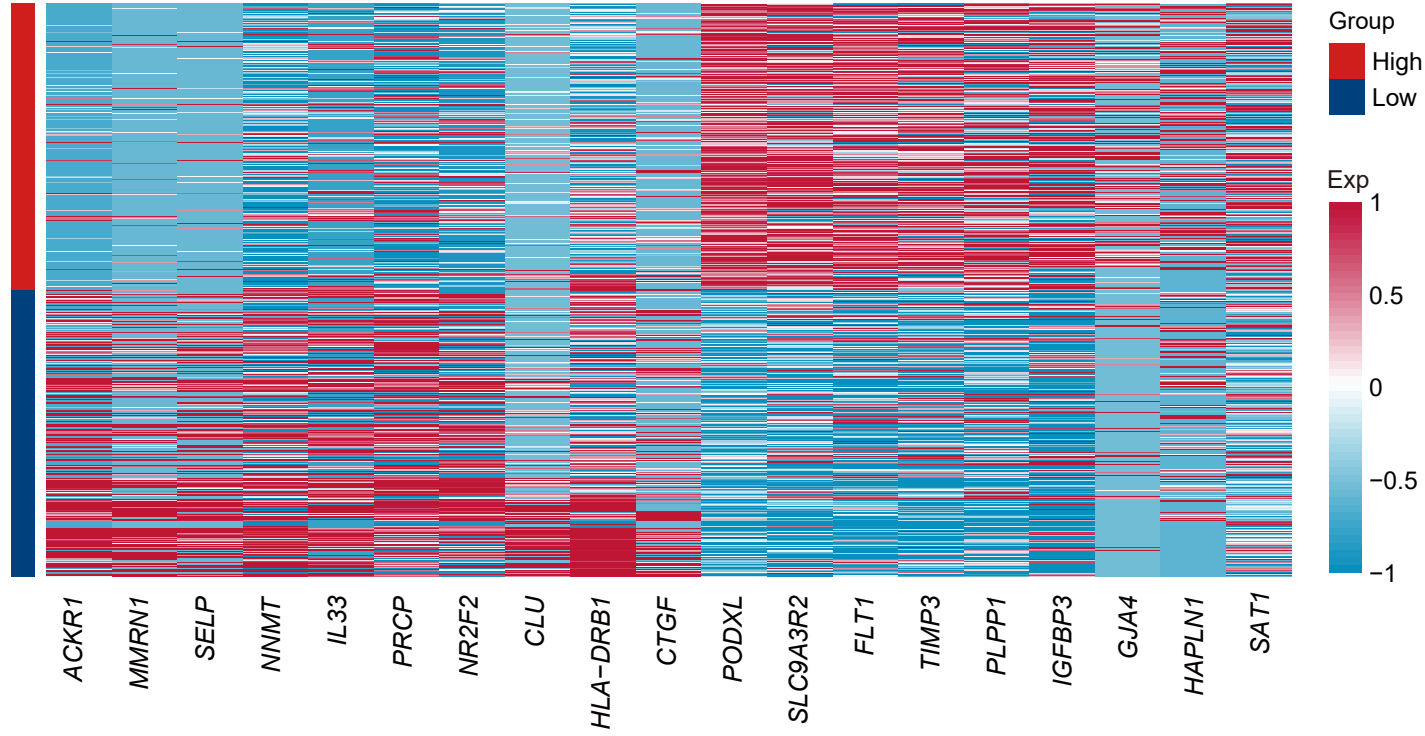

Supplement: Supplementary file 3 — Supplementary Material 3: Figure S3. Heatmap showing the differentially expressed genes between PODXL high TECs and PODXL low TECs in scRNA-seq data. TECs, tumor endothelial cells; scRNA-seq, single-cell RNA sequencing. [file 40364_2024_655_MOESM3_ESM.pdf]

A

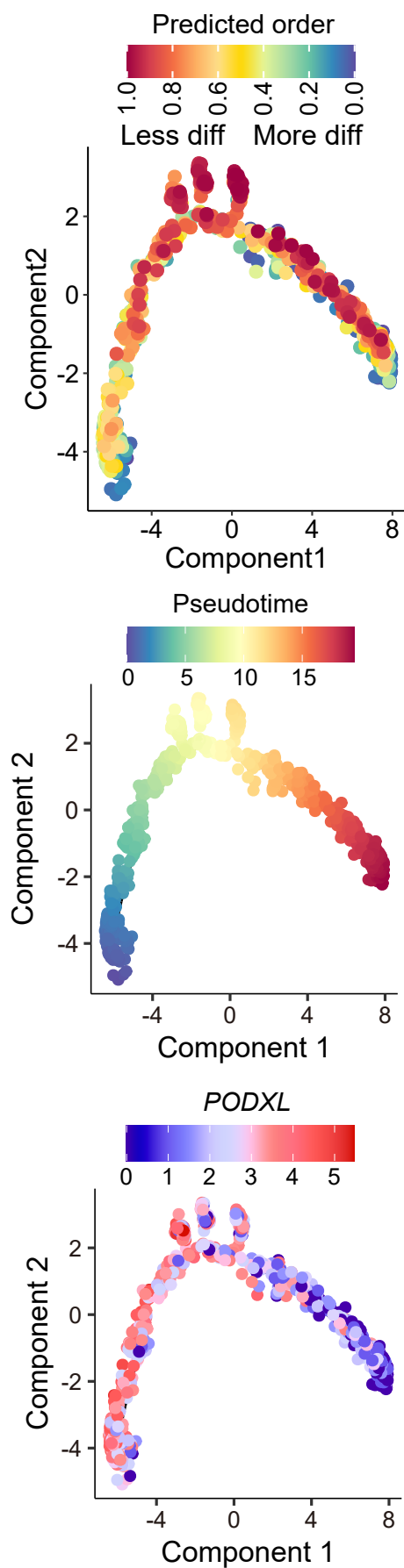

B

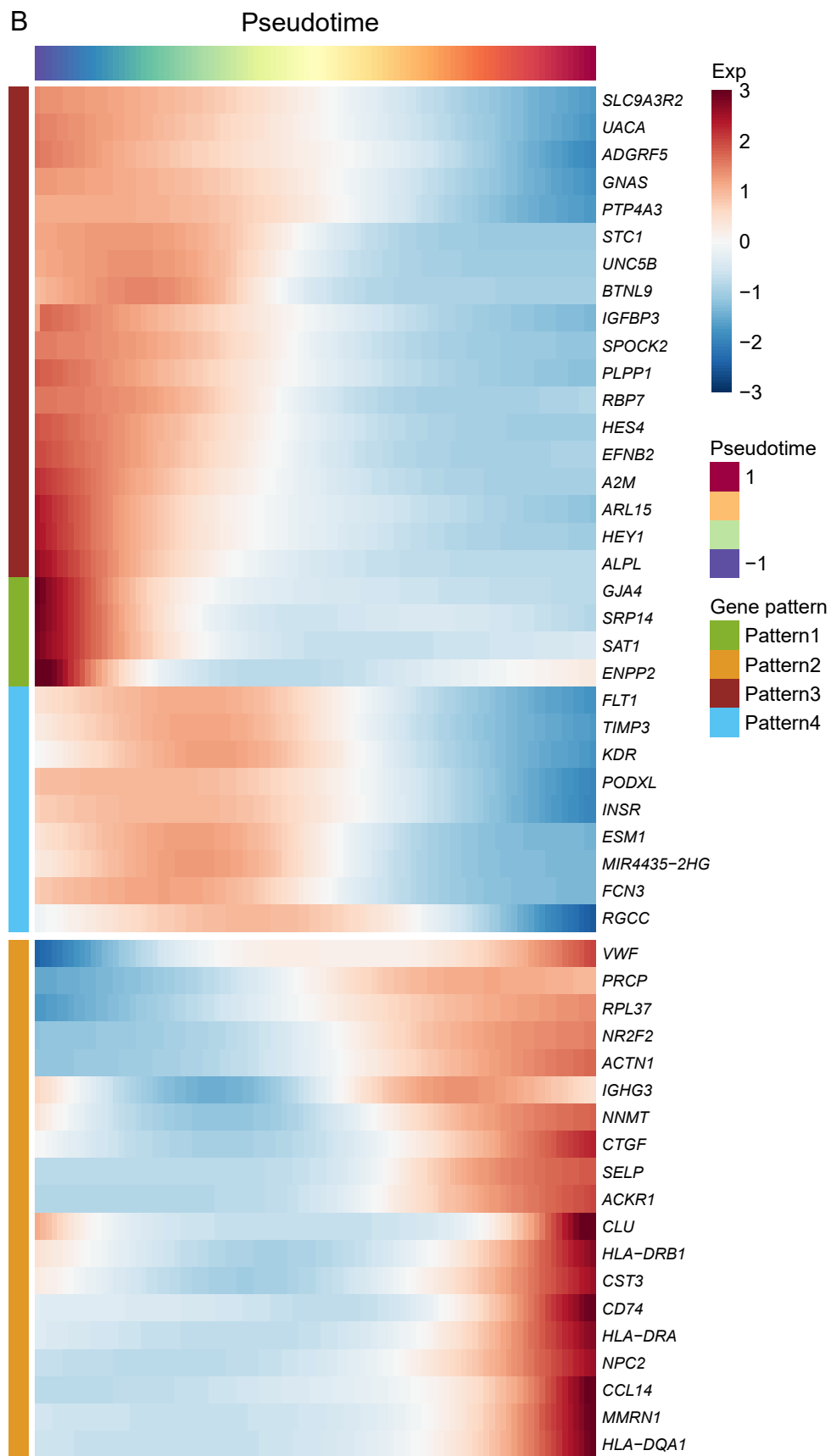

Supplement: Supplementary file 5 — Supplementary Material 5: Figure S5. Pseudotime analysis of PODXL low and PODXL high TECs in scRNA-seq data. (A) Three trajectory plots showing the predicted order of cell differentiation, pseudotime, and the expression levels of PODXL . (B) Heatmap showing the dynamic expression patterns of different genes along the pseudotime trajectory. Genes are categorized into four distinct expression patterns, marked by different colors. [file 40364_2024_655_MOESM5_ESM.pdf]

A

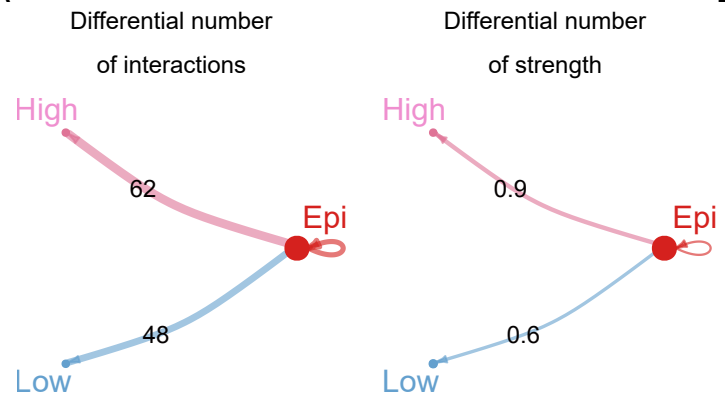

B

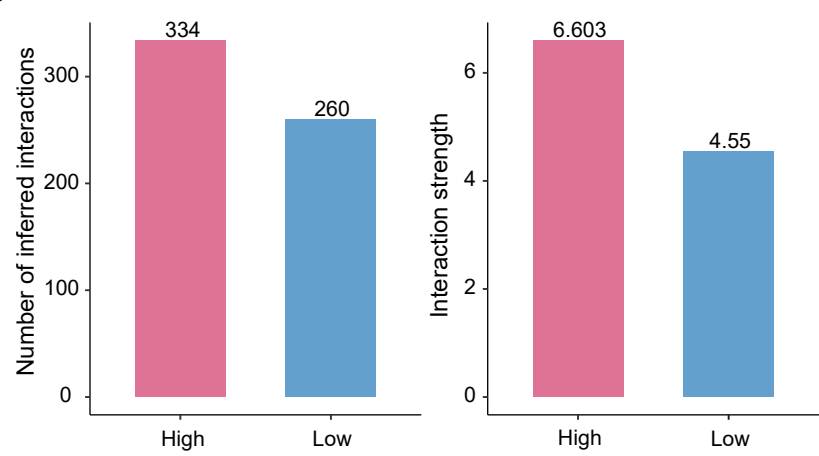

C

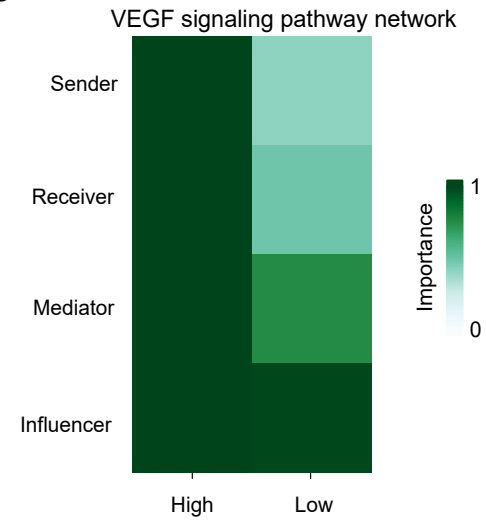

D

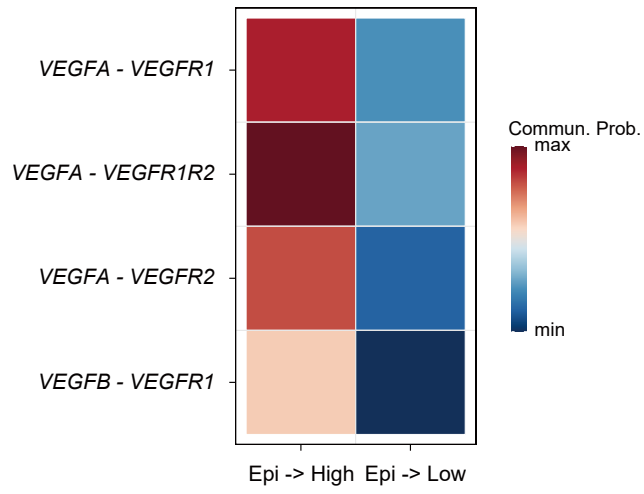

E

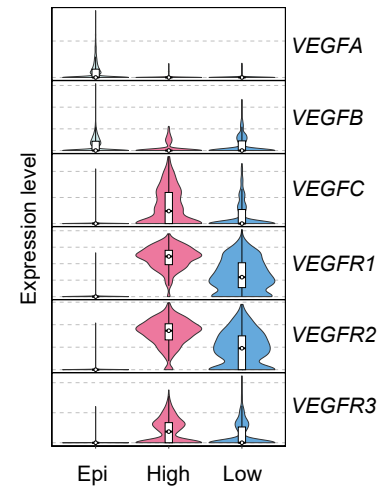

Supplement: Supplementary file 6 — Supplementary Material 6: Figure S6. Cell communication analysis of PODXL low and PODXL high TECs with epithelial cells. (A) Differential interaction network illustrating the number of interactions and interaction strength between PODXL low and PODXL high TECs and epithelial cells. The numbers indicate the count of differential interactions/strength. (B) Bar charts of interaction metrics. Left: Number of inferred interactions. Right: Interaction strength. (C) Heatmap showing the importance of different cell roles (Sender, Receiver, Mediator, Influencer) in the VEGF signaling pathway. Darker green indicates higher importance. (D) Heatmap showing the maximum communication probability for different VEGF ligand-receptor pairs. Columns represent the direction of communication. (E) Violin plots showing the expression levels of VEGF ligands and receptors. VEGF: Vascular endothelial growth factor. [file 40364_2024_655_MOESM6_ESM.pdf]

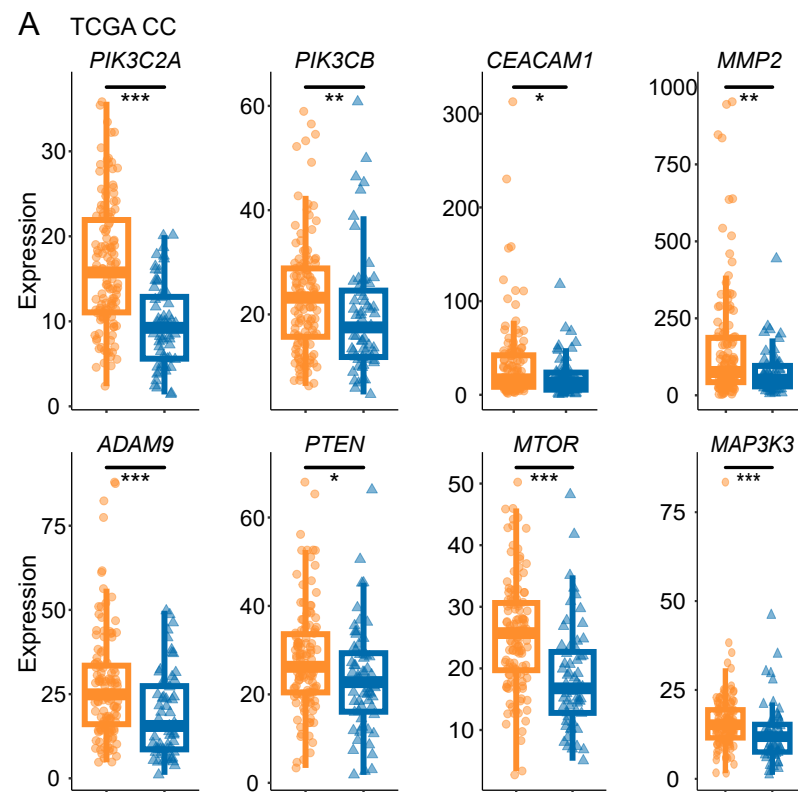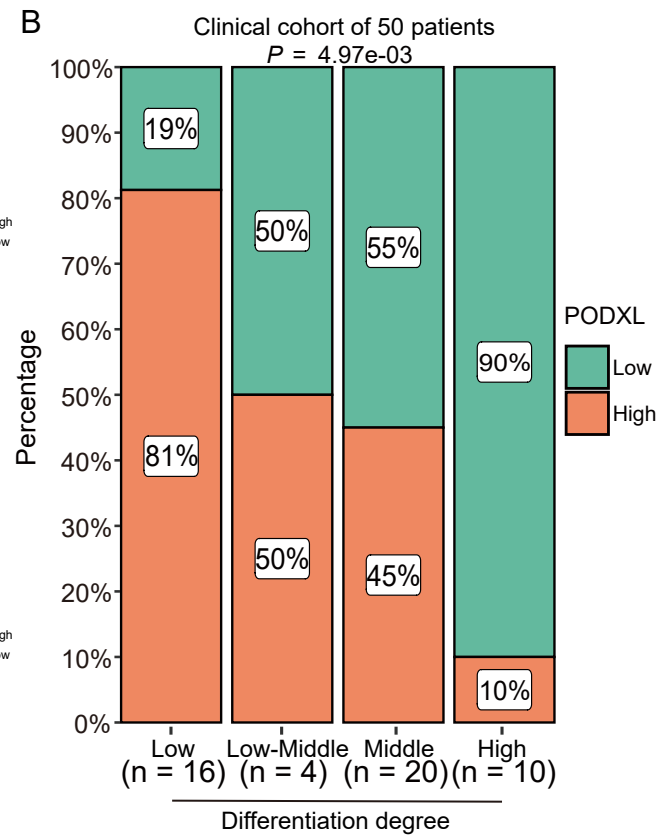

**C** Expression of Ki67 (PODXL High)

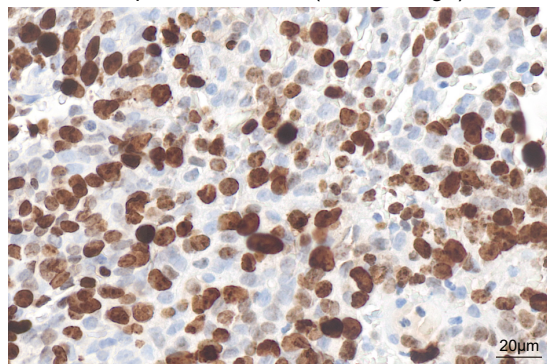

Expression of Ki67 (PODXL Low)

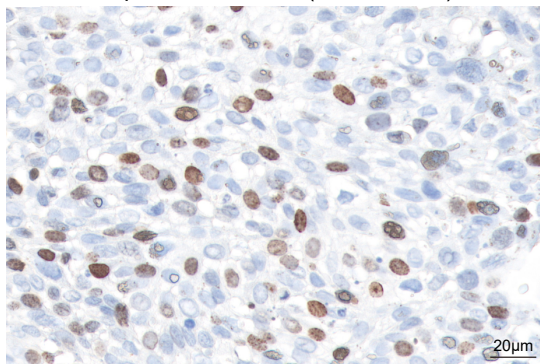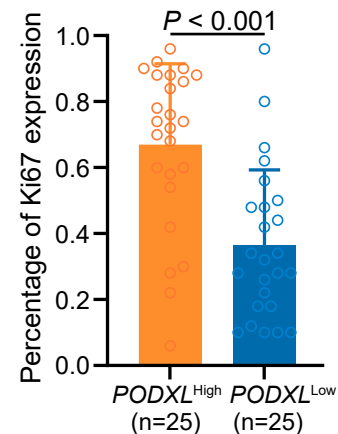

Supplement: Supplementary file 8 — Supplementary Material 8: Figure S8. Analysis of PODXL expression in relation to epithelial cell differentiation and migration. (A) Box plots displaying the expression levels of genes associated with epithelial proliferation, invasion and metastasis in PODXL high and PODXL low groups from the TCGA dataset. (B) Percentage bar chart showing the distribution of differentiation degrees (Low, Low-Middle, Middle, High) in our clinical cohort of 50 patients stratified by PODXL expression levels. (C) Immunohistochemistry analysis of Ki67 expression, comparing the percentage of Ki67-positive cells in PODXL high and PODXL low groups, with representative images and quantification. *, p < 0.05; **, p < 0.01; ***, p < 0.001 (Wilcoxon test). [file 40364_2024_655_MOESM8_ESM.pdf]
