# Supplementary material for: Tumor endothelium-derived PODXL correlates with immunosuppressive microenvironment and poor prognosis in cervical cancer patients receiving radiotherapy or chemoradiotherapy
Source: Biomark Res. 2024 Sep 18;12:106. doi: 10.1186/s40364-024-00655-0 (PMC11409751; doi:10.1186/s40364-024-00655-0)
Supplement: Supplementary file 4 — Supplementary Material 4: Figure S4: Gene set variation analysis revealed the comparation of tumor pathways between the PODXL low and PODXL high TECs in scRNA-seq data. ***, p < 0.001 (Wilcoxon test). [file 40364_2024_655_MOESM4_ESM.pdf]

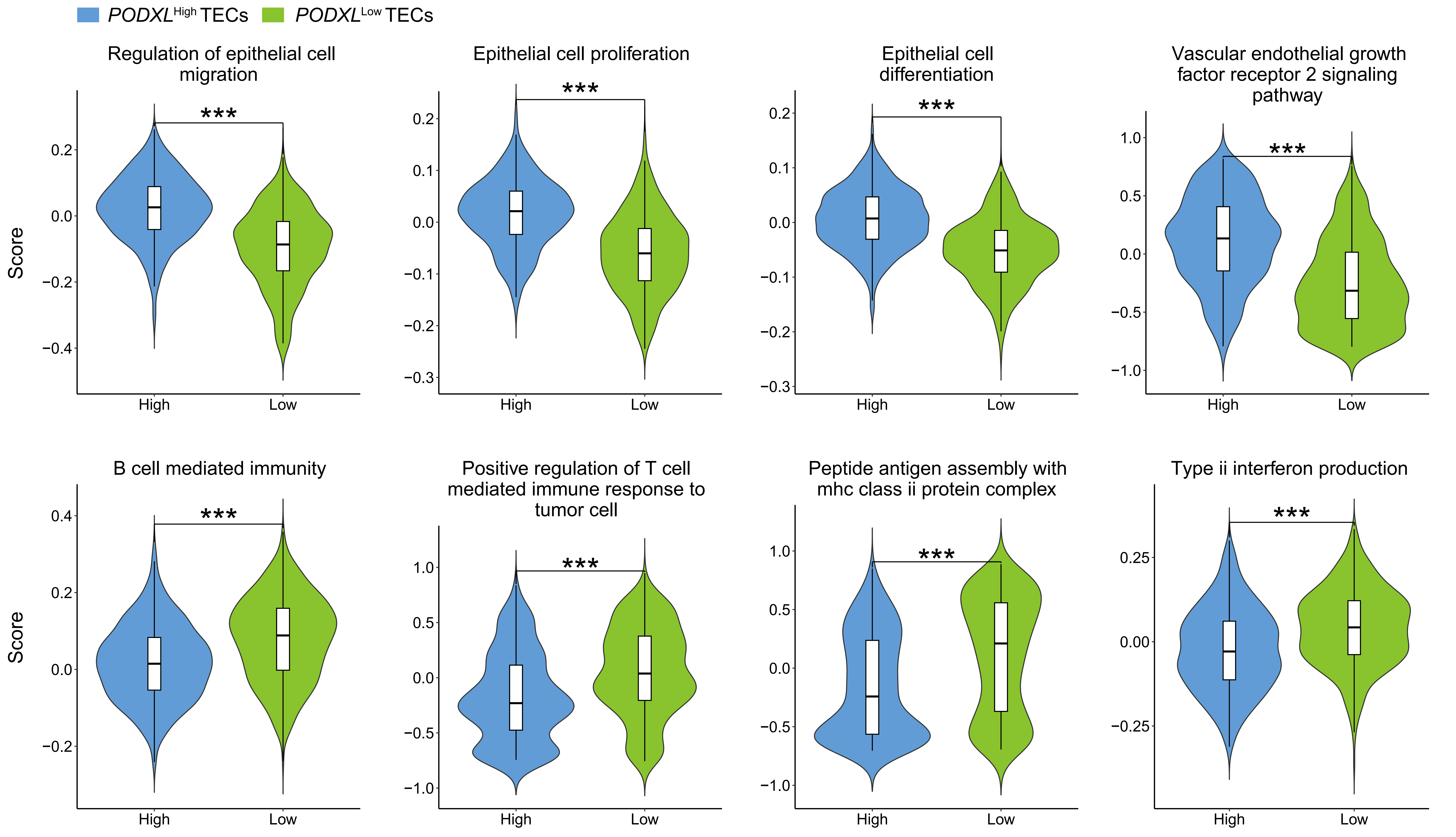
PODXL^High TECs
PODXL^Low TECs
Regulation of epithelial cell migration
Epithelial cell proliferation
Epithelial cell differentiation
Vascular endothelial growth factor receptor 2 signaling pathway
B cell mediated immunity
Positive regulation of T cell mediated immune response to tumor cell
Peptide antigen assembly with mhc class ii protein complex
Type ii interferon production
Score
High
Low
***
