# Supplementary material for: Tumor endothelium-derived PODXL correlates with immunosuppressive microenvironment and poor prognosis in cervical cancer patients receiving radiotherapy or chemoradiotherapy
Source: Biomark Res. 2024 Sep 18;12:106. doi: 10.1186/s40364-024-00655-0 (PMC11409751; doi:10.1186/s40364-024-00655-0)
Supplement: Supplementary file 7 — Supplementary Material 7: Figure S7. The feature of PODXL high CC groups in TCGA database. Gene set enrichment analysis showing the enriched pathways in the PODXL high group. NES: normalized enrichment score. [file 40364_2024_655_MOESM7_ESM.pdf]

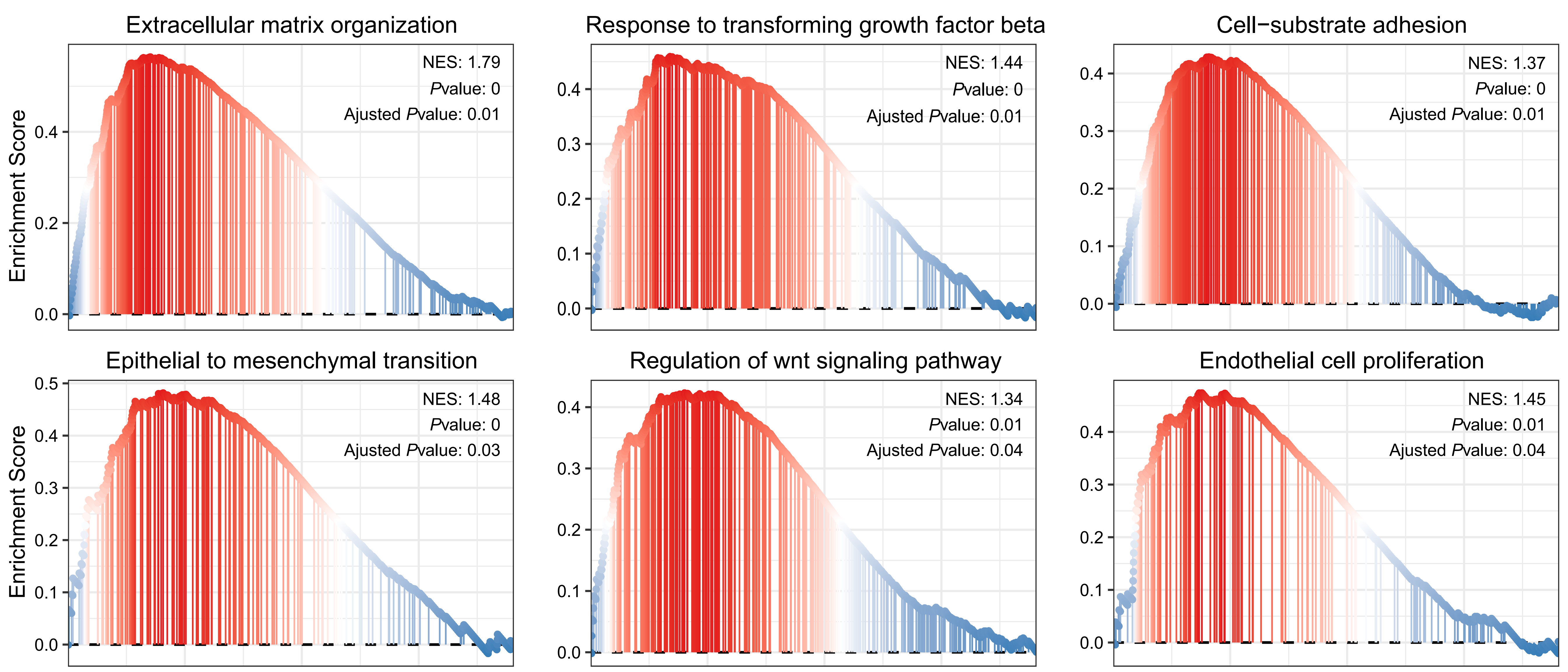
Extracellular matrix organization
NES: 1.79
Pvalue: 0
Ajusted Pvalue: 0.01
Response to transforming growth factor beta
NES: 1.44
Pvalue: 0
Ajusted Pvalue: 0.01
Cell−substrate adhesion
NES: 1.37
Pvalue: 0
Ajusted Pvalue: 0.01
Epithelial to mesenchymal transition
NES: 1.48
Pvalue: 0
Ajusted Pvalue: 0.03
Regulation of wnt signaling pathway
NES: 1.34
Pvalue: 0.01
Ajusted Pvalue: 0.04
Endothelial cell proliferation
NES: 1.45
Pvalue: 0.01
Ajusted Pvalue: 0.04
Enrichment Score
Enrichment Score
