# Supplementary material for: Tumor endothelium-derived PODXL correlates with immunosuppressive microenvironment and poor prognosis in cervical cancer patients receiving radiotherapy or chemoradiotherapy
Source: Biomark Res. 2024 Sep 18;12:106. doi: 10.1186/s40364-024-00655-0 (PMC11409751; doi:10.1186/s40364-024-00655-0)
Supplement: Supplementary file 9 — Supplementary Material 9: Figure S9. The difference of immune infiltration between the PODXL low and PODXL high CC groups in TCGA database. *, p < 0.05; **, p < 0.01; ***, p < 0.001 (Wilcoxon test). [file 40364_2024_655_MOESM9_ESM.pdf]

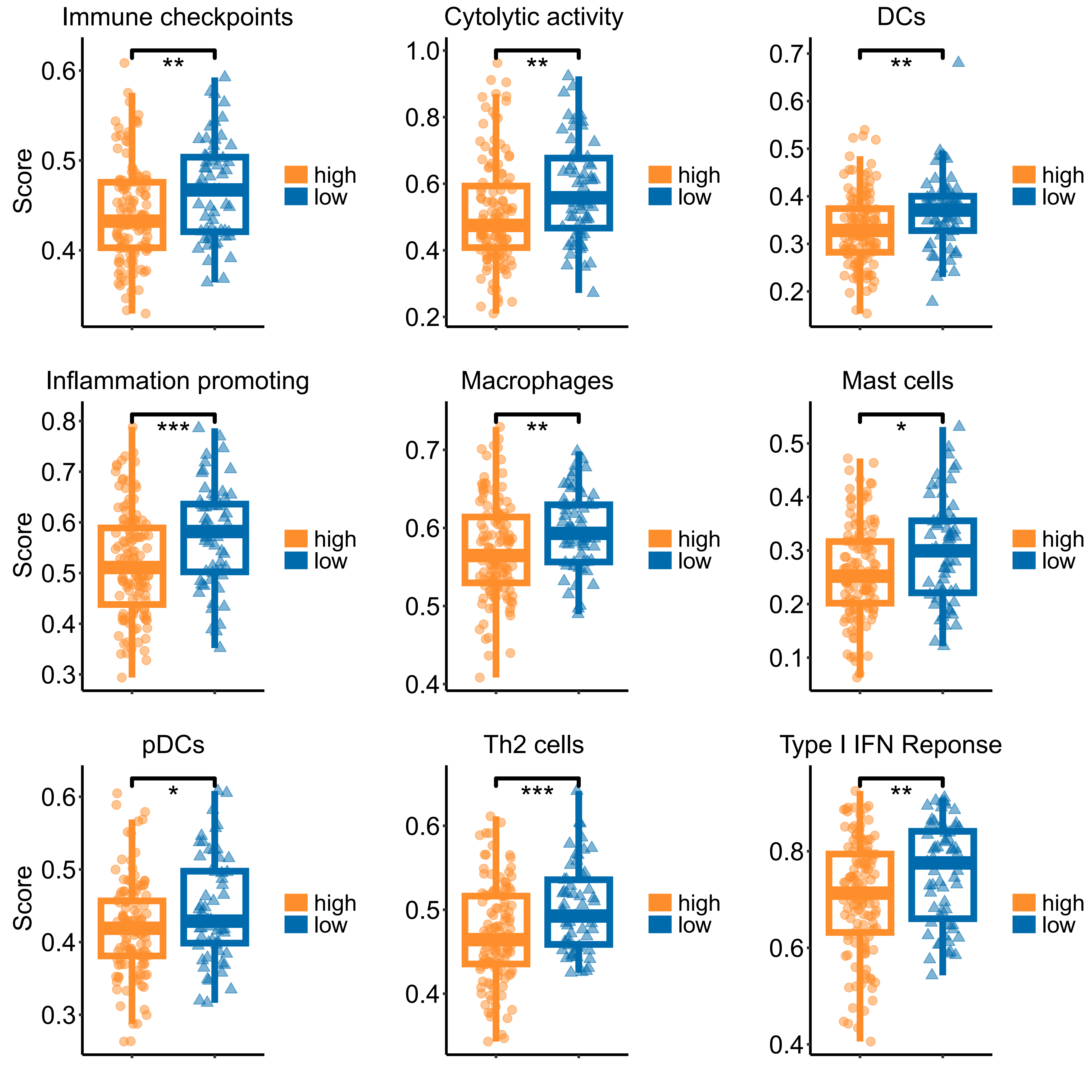
Immune checkpoints
Cytolytic activity
DCs
Inflammation promoting
Macrophages
Mast cells
pDCs
Th2 cells
Type I IFN Reponse
Score
high
low
**
***
*
